# Community voices: Achieving real diversity in STEM requires the ability to transform institutions

**DOI:** 10.1038/s41467-021-27376-4

**Published:** 2022-03-25

**Authors:** Jory C. Lerback, Monique M. Holt, Gabriela A. Enriquez St. Pierre, Annie Putman, Crystal L. Tulley-Cordova, William A. Smith, Liliana Caughman, Stephanie Alvarez

**Affiliations:** 1 Department of Geology and Geophysics, University of Utah, Salt Lake City, UT 84112 USA; 2 Department of Earth, Planetary and Space Sciences, University of California Los Angeles, Los Angeles, CA 90095 USA; 3 Department of Earth and Environmental Sciences, University of Illinois Chicago, Chicago, IL 60607 USA; 4 Navajo Nation Department of Water Resources, P.O. BOX 678, Fort Defiance, AZ 86504 USA; 5 Department of Education, Culture, & Society, University of Utah, Salt Lake City, UT 84112 USA; 6 Division of Ethnic Studies, University of Utah, Salt Lake City, UT 84112 USA; 7 Native Environmental Science Department, Northwest Indian College, Bellingham, WA 98226 USA; 8 Institute for Sustainable Solutions, Portland State University, Portland, OR 97201 USA; 9 School of Life Sciences, Earth Systems Science for the Anthropocene, Arizona State University, Tempe, AZ 85281 USA; 10 Facultad de Finanzas, Gobierno y Asuntos Internacionales, Universidad Externado de Colombia, Bogotá, 111711 Colombia

**Keywords:** Policy, Communication, Science in culture, Education

## Abstract

Resilience is often invoked to address systemic marginalization (e.g. racism) in academia but inadvertently maintains harmful systems. We argue that the ability to transform systems, as opposed to persevering within them, must be prioritized to make real, lasting change.

## Resilience requires a thoughtful definition

Academia and especially STEM disciplines must achieve diversity, equity, justice, and inclusion (DEJI), but we argue that irresponsible resilience-rhetoric hinders or halts progress. While there are multiple definitions of resilience, some limit the focus to individual qualities that implicitly maintain the status quo. We propose other definitions have the power to enact lasting, systemic change^1–3^.

The most colloquial definition of resilience is the ability to recover after experiencing difficulties. This can be likened to perseverance, grit, and bravery, traits that are not inherently problematic but that we believe are disproportionately expected of scientists belonging to marginalized groups who exist in hostile work environments. It is our opinion that it is irresponsible of people in positions of power to use this definition because it fails to hold the social systems, the source of the hostility, accountable. This definition prompts only individual-focused corrective actions (e.g., mentoring) and measures success narrowly^4,5^ (e.g., H-index). We (the authors, who are scientists that hold intersecting marginalized identities) object to our career success depending on our personal ability to withstand identity-based hardships. We call for systemic changes, which means changing the work environment to become more inclusive, equitable, and welcoming for everyone, irrespective of their identities. In this essay, we discuss marginalization in terms of the intersecting dimensions of race, ethnicity, gender, sexuality, religion, nationality, physical ability, social standing, and many others.

We do not call for the end of resilience; it is a useful concept. Indeed, the other most common definition of resilience is the ability of something (in this case, people) to return to an original state after experiencing stress is less prescriptive and can result in positive outcomes for marginalized groups in a changing, imperfect system. However, this too must be used with cares—the original way of being must explicitly foster marginalized individuals’ agency^6^. For example, our self-identified, desired, original state includes being happy and supported. In our work environments, this presents as freely and safely engaging in creative research without artificial barriers to success where we will not be in danger of psychosocial burnout.

Achieving the latter definition of resilience requires assessing the inequalities and injustices that undergird institutions, a critical systemic context. Encouraging resilience without this context reinforces the problematic status quo. While work stress and difficult situations are arguably unavoidable, studies suggest those harms disproportionately impact marginalized people^7–9^, and underlying this is a myriad of additional identity-based burdens (microaggressions, explicit biases, discriminatory practices, etc.). For example, racism can have an acute and immediate negative impact, and the associated chronic stress leads to serious psychological and physiological harms. This condition has been identified as racial battle fatigue, encapsulating the negative psychological, emotional, and physiological impacts of systemic racism and microaggressions^10,11^. So, without large-scale cultural and policy change to make healthier environments, we argue that individual resilience is limited by these cumulative harms.

## A four-part framework

Consequently, it is imperative to understand how resilience can prioritize marginalized individuals and groups. We combine transdisciplinary perspectives on resilience drawing from psychology, socio-ecology, systems theory, and engineering^12–17^. We re-apply these perspectives to DEJI in social systems and work environments. These perspectives shift the burden of being resilient away from individuals. This resilience framework consists of four capacities for responding to systemic stress: absoptive capacities, adaptive capacities, restorative capacities and transformative capacities. We hope this organizing structure will be useful to policymakers, activists, and other educational leaders.

First, despite stressful situations, a person may want to stay in their job, degree program, or laboratory. To achieve this goal, it is pertinent to develop absorptive capacity. Absorptive capacity refers to the ability to respond to stress, adversity, or change by focusing on one’s outlook or behaviors or finding support through social connections^18,19^. Supportive communities such as DEJI clubs, LGBTQ+ Resource centers, or inclusive research groups, facilitate community building, which helps to strengthen this capacity^5^. Often, we feel understood, relieved, and a sense of community by sharing identity crises, imposter syndrome, and other worries in a respectful and trusting environment. Organizations can help their members build absorptive capacity by facilitating financial support for counseling services and helping newer students navigate the opaquer aspects of graduate school.

Sometimes the stress, damage, and burden of one’s job or program may be too much and lead to making a career move. This looks like finding a new research group or even leaving STEM altogether. Adaptive capacity allows individuals to make changes to their circumstances that best accelerate them towards their original state.

A second capacity in the proposed framework, adaptive capacity, refers to the ability to change one’s circumstances or role to respond to stress, adversity, or change^20^. Professional societies (like the American Indian Science Engineering Society or GeoLatinas) promote this by fostering collaborative environments and mentorship across institutions. Adaptive responses to stress can look like changing career paths (e.g., industry vs. academia) or switching degree programs, provided these changes move individuals towards their original state. Organizations can help people build adaptive capacity by supporting the membership of professional societies and encouraging members to form external collaborative relationships.

Stressful and unsafe work environments take their toll on people’s physical and mental health. Whether one perseveres or changes their situation, it is important to heal and be cognizant of potentially harmful situations or environments in the future, to avoid additional stress and damage associated with racial battle fatigue and role strain^21^. In reality, this physical and emotional harm prevents people from returning to their unscathed state, thus requiring the strengthening of one’s restorative capacity.

The third capacity in our resilience framework, restorative capacity, refers to the process of recognizing situations where stress, harm, and change will happen, and taking steps to take care of one’s self and community^22,23^. This pillar of resilience is already a part of some traditional cultural practices. Affinity groups practice sharing personal experiences so marginalized individuals and groups can learn to anticipate, recognize, and develop healing and preventative measures associated with—isms and—phobias. For example, Diné (Navajo) have a ceremony called Hozhoji, which helps people refocus their being if their life gets out of balance^24^. Organizations can help strengthen restorative capacity by encouraging member participation in affinity groups, wellness discussions about culture and privilege, or other activities designed to foster an inclusive environment.

We argue that the additional work that marginalized individuals and groups must do to build any of these capacities (as they often do not already exist in STEM settings) is an unfair and unjust burden. These capacities accept the inequitable status quo and fail to address symptoms of the larger problem: the exclusive nature of STEM institutions. This brings us to the final component of resilience.

The final capacity in our framework, transformative resilience, refers to the ability to shift social structures, expectations, and rules to address underlying causes of stress, adversity, and harm^16,25^. On a personal level, transformative capacity includes advocacy, activism, and clear communication of needs. The STEM community has taken strides towards transformative capacity, from academic institutions waiving Graduate Record Examinations requirements to journal publications providing a platform for anti-racist STEM policy suggestions. For institutions, transformative capacity requires sustained listening, reorganization, co-designing inclusive policy, and adequate financial compensation at every level. First steps towards building transformative capacity can include funneling resources (i.e., money, space, equipment), building formal and informal coalitions that give marginalized individuals and groups powerful voices, and physically changing institutional spaces to reflect decolonized ideologies^26^. Transformative resilience prioritizes marginalized communities and preserves their autonomy, and includes the larger STEM structures and community.

## Utilizing resilience

This transdisciplinary resilience framework helped us envision the different ways in which DEJI policy can be approached. In our experience, capacities 1–3 are non-comprehensive as they benefit individuals on a case-by-case basis. For example, groups may unintentionally focus solely on absorptive capacities by teaching members to work within established workplace cultures. While useful, these strategies do not necessarily integrate the other capacities or empower members to rectify systemic inequities for future generations. Capacities 1–3 address diversity and inclusion by increasing perseverance within the current system, with success measured as the retention of minoritized individuals. However, other scholars and we have observed a conflict between “perseverance” (capacities 1–3) and transformation; the better you work within a system, the less incentive and energy you have to change it^2,16,17^. We argue that only prioritizing transformative resilience can lead to true equity and justice.

To start the shift to transformative resilience, we argue that organizations should create inclusive listening spaces for the co-development of DEJI culture and policy, incrementally build trust through transparency and action, as well as (equitably) provide resources for building individual resilience. Further guidance on this process and other theories of change can be drawn from sociology, organization studies, restorative justice, and political science to construct authentic, systemic change^27–31^.

We urge the readers to review or develop both their personal and their organization’s DEJI action plan through this systems-oriented, accountable, and responsible lens of resilience. This means clarifying the processes through which people holding marginalized identities can co-develop and co-design STEM and cultivating our ability to do so.

## Notes

**Peer review information:** *Nature Communications* thanks Molly Carnes and Sapna Cheryan for their contribution to the peer review of this work.
